# Disseminated Infection Caused by Novel Species of *Microsporidium*, Thailand

**DOI:** 10.3201/eid1802.111319

**Published:** 2012-02

**Authors:** Chusana Suankratay, Ekkachai Thiansukhon, Voraphoj Nilaratanakul, Chaturong Putaporntip, Somchai Jongwutiwes

**Affiliations:** Chulalongkorn University, Bangkok, Thailand

**Keywords:** microsporidia, myositis, Endoreticulatus, lepidoptera, parasites, Thailand

## Abstract

We describe a case of microsporidial myositis in a healthy man from Thailand. The small subunit rRNA sequence of this microsporidium is novel and has a close phylogenetic relationship with *Endoreticulatus*, a genus of lepidopteran microsporidia. Myositis could be caused by more genera of microsporidia than previously known.

Microsporidia are obligate intracellular eukaryotes of broad host range (*1*). At least 15 species have been implicated in human infections. Although few microsporidiosis cases have been reported, they have emerged as opportunistic infections in patients with HIV infection (*1*). In addition, microsporidiosis has increasingly been diagnosed in other immunocompromised patients, including those who have received an organ transplant, and in immunocompetent persons (*1*). Various clinical manifestations, ranging from localized (diarrhea, cholangitis, sinusitis, keratitis) to disseminated (myositis, osteomyelitis, encephalitis) infection have been observed, depending on the causative agent and host immune status (*1*). We report microsporidial myositis caused by a suspected new species of *Microsporidium* in an otherwise healthy man.

## The Study

A 43-year-old man (a welder living in Lopburi Province, central Thailand) sought treatment at King Chulalongkorn Memorial Hospital, Bangkok, on November 30, 2010. He had experienced difficulty in swallowing for 2 weeks. Fifteen months before hospital admission, he experienced a low-grade fever and generalized muscle pain, especially in his lower back and both thighs. Despite several courses of medication, hospital, his condition had not improved.

Ten months before admission, weakness of lower and upper limbs developed in his proximal muscle, primarily on the left side. Two months before admission, his entire left leg was swollen, and he was confined to bed. During this illness, he had lost ≈29 pounds. He reported no travel abroad and had not taken any herbal medications. He did not smoke, drank alcohol occasionally, and did not use illicit drugs. He had no history of recurring infections during childhood and early adulthood.

Physical examination showed no keratoconjunctivitis, but indicated engorged jugular veins and right ventricular heave with loud pulmonic valve closure sound. Abdominal examination showed no hepatosplenomegaly. Non-pitting edema of the entire left leg was observed. Neurologic examination showed hyporeflexic proximal muscle weakness of grade III–IV/V in all limbs. Complete blood count showed the following: hematocrit 43.5%, leukocyte count 5,230/mm^3^ (79% neutrophils, 14% lymphocytes, 4% eosinophils, and 3% monocytes), platelet count 209,000/mm^3^. Liver function test showed the following results: total bilirubin 0.34 mg/dL, alkaline phosphatase 57 U/L (reference value 42 U/L–121 U/L), aspartate transaminase 268 U/L (4 U/L–36 U/L), alanine transaminase 101 U/L (4 U/L–36 U/L), albumin 3.1 g/dL, and creatine phosphokinase 4,308 U/L (60 U/L–400 U/L). Test results were negative for antibody against nuclear antigen and HIV (2 tests, 1 month apart). CD4 cell count was 368/μL (24%), and serum protein electrophoresis showed polyclonal gammopathy.

Chest radiograph showed pulmonary hypertension without significant pulmonary infiltration. Severe pulmonary hypertension without left ventricular dysfunction or left-to-right shunt was evident on electrocardiogram. Computed tomographic scan showed an enlarged pulmonary trunk (4 cm in diameter) and right and left main pulmonary arteries (thrombosis was not shown in the lumens). Electromyograph showed irritative myopathy without evidence of large-fiber sensory and motor polyneuropathy.

**Figure 1:**
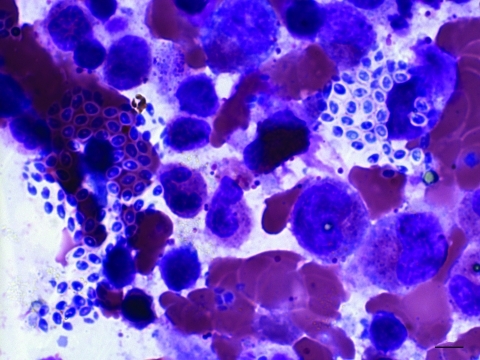
*Microsporidium* species from bone marrow aspiration specimen (Wright stain) from a 43-year-old man, Thailand. The image shows focal aggregations of microsporidia-like microorganisms with a belt-like stripe (1.0–1.5 μm x 1.2-2.2 μm). Original magnification ×100.

A biopsy of left deltoid and both vastus lateralis muscles showed necrotizing granulomatous inflammation without any organisms by Gram, acid-fast bacilli, Wright, and Gomori methenamine silver staining. Bone marrow biopsy showed normal cellularity with increased plasma cells and histiocytes and focal aggregation of microsporidial spores with characteristic belt-like stripe (Figure 1). A 24-hour urine sample (centrifuged) yielded characteristic microsporidial spores on modified trichrome stain, 1.0–1.5 μm × 1.2–2.2 μm, similar to those observed in the bone marrow sample. Because of an overgrowth of *Candida* yeast*,* we did not isolate the organism from the urine sample.

**Figure 2:**
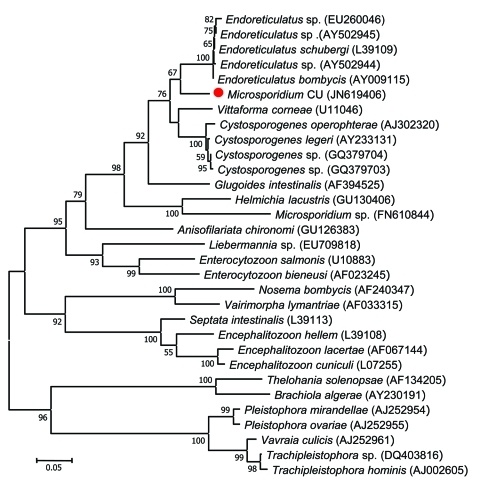
Phylogenetic tree inferred from the small subunit rRNA sequences of microsporidia in this study and those in the GenBank database by using the maximum-likelihood method as implemented in MEGA5.05 software (*2*). Red circle indicates a novel microsporidium identified in this study (Microsporidium CU) that caused myositis. GenBank accession numbers are listed in parentheses after each species. Bootstrap percentages >50% based on 1,000 replicates are shown on the branches. The tree is drawn to scale, with branch lengths measured in the number of nucleotide substitutions per site.

Analysis of the small subunit rRNA gene spanning 797 bp of DNA from microsporidial spores isolated from this patient showed a novel sequence (GenBank accession no. JN619406). Phylogenetic reconstruction using the maximum-likelihood method placed the patient’s *Microsporidium* sp. within the cluster of the genera *Endoreticulatus*, *Cystosporogene,* and *Vittaforma* (Figure 2) (*2*). This species has a close phylogenetic relationship with *Endoreticulatus* spp. Notably, the guanine-cytosine content of the SSU rRNA locus of microsporidia within the genus *Endoreticulatus* varied from 51.4% to 51.8%, with the base composition distance 0.001–0.029. However, the small subunit rRNA sequence of microsporidium from our patient (GenBank accession no. JN619406) contained 52.4% guanine-cytosine, and the base composition distance differed from *Endoreticulatus* spp. from 0.094–0.170, making it unlikely that the patient was infected with an organism from this genus*.*

The patient was treated with albendazole (800 mg/d continuously). Fever subsided after 1 week of treatment, and weakness gradually improved. He was discharged on a regimen of oral albendazole (800 mg/1×/d) but died unexpectedly from aspiration pneumonia 1 month later. An autopsy could not be performed.

## Conclusions

Microsporidia can cause either localized or disseminated infection in humans. The true prevalence of microsporidiosis in humans may be underestimated because of asymptomatic infection in most healthy persons, clinician unawareness, and difficulties in diagnosis. Myositis caused by microsporidia is rare; only 12 patients (including our patient) have been reported in the literature (Table A1; *3**–**12*). Among these 12 patients, 9 were male and 3 were female (age range 4 months to 67 years). Patients were from all continents except Europe: United States (7 patients), Australia (2), Haiti (1), the Gambia (1), and Thailand (1). The patient described here is the first report from Asia. *Trachipleistophora* sp. (6 patients) is the most common causative agent of myositis, followed by *Brachiola* sp. (3 patients), *Pleistophora* sp., *Tubulinosema acridophagus* sp., and *Microsporidium* spp. (1 patient each). However, the species of microsporidia originally described in 2 reports (*5**,**6*) was changed from *Pleistophora* sp. to *Trachipleistophora* sp. upon reexamination of the ultrastructures by electron microscopy.

Although ultrastructural study of the microsporidium from this patient has not been performed, the SSU rRNA sequence clearly supports the finding that it is a novel species, closely related to *Endoreticulatus* spp. (Figure 2). Most patients in previous reports had disseminated infection or isolated myositis. The patient described here had pulmonary hypertension, likely caused by microsporidia; several reports have shown that these organisms can cause pulmonary infection (*13*).

The routes of microsporidial infection are still uncertain, but the species that can infect humans have been identified in water sources and in farm, domestic, and wild animals. Furthermore, *Trachipleistophora* spp. have been found only in human hosts. However, on the basis of the feature of bisporous sporophorous vesicles, *T. anthropophthera* was proposed to be related to *Telomyxa* spp., which are parasites of mayflies. *T. anthropophthera* was reported to be the causative agent of keratitis in an HIV-infected patient from Thailand (*14*). *Brachiola* spp. (formerly *Nosema* spp.) are known to be pathogens of several kinds of insects, including mosquitoes (*10*). *Pleistophora* spp. have been found in fish and reptiles, but *P. ronneafiei* has never been identified in any hosts other than humans. *Microsporidium* spp. that can infect humans are *M. ceylonensis* and *M. africanum*. Both species were reported from the patients with keratitis from Sri Lanka and Africa, respectively, although their natural hosts remain unknown.

To our knowledge, the microsporidium possessing a novel SSU rRNA sequence closely related to *Endoreticulatus* spp. has never been identified in patients with myositis. Notably, certain lepidopteran microsporidia can produce various responses in nontarget hosts, from refractory to severe infections. Although we have no evidence regarding the route or source of this patient’s infection, the close phylogenetic relationship of this microsporidium and lepidopteran microsporidia suggests that these non–blood-sucking insects could be natural hosts. Water or foods contaminated with lepidopteran insects could not be excluded as the source of infection. Likewise, recent reports that *Trachipleistophora extenrec* can cause myositis in anteaters (*15*) and that *Tubulinosema* sp. can cause myositis in an immunosuppressed patient (*12*) further support the role of insect microsporidia as human pathogens.

## Appendix

**Table A1:** Characteristics of 12 patients with microsporidial myositis*

Patient no.	Age/sex	Country	Year	Clinical features	Illness duration	Tissues and organs involved	HIV status	CD4 count/μL	Species	Treatment	Outcome
1	4 mo/M	United States	1973	Fever, weakness	4 mo	Heart and striated muscle	NA	NA	*Brachiola connori*	NA	Died
2	20 y/M	United States	1985	Fever, weakness, lympadenopathy, testicular atrophy, gynecomastia, sinusitis	7 mo	Striated muscle	–	NA	*Pleistophora raronneafiei*	CTX	Survived >4 mo
3	33 y/M	Haiti	1993	Fever, weakness, weight loss, co-infected with *Norcardia* sp.	2 mo	Heart and striated muscle	AIDS	NA	*Trachipliestophora* sp.	PYM	Died
4	35 y/M	The Gambia	1996	Fever, weakness, anemia	NA	Striated muscle	AIDS	57	*Trachipliestophora* sp.	CLI	NA
5	34 y/M	Australia	1996	Fever, weakness, keratitis, sinusitis	3 mo	Striated muscle, cornea, sinus	AIDS	6	*T. hominis*	NA	Partial recovery; died of HIV infection
6	33 y/M	United States	1996	Fever, hemiparesis, seizures	2 mo	Heart, striated muscle, brain, bone, viscera	AIDS	2	*T. anthropophthera*	ABZ, PYM, SDZ	Died
7	8 y/F	United States	1996	Weakness, seizures, aphasia, dyspnea	4 mo	Heart, striated muscle, brain, bone, viscera	AIDS	NA	*T. anthropophthera*	Agents for toxoplasmosis	Died
8	31 y/M	United States	1998	Fever, weakness	5 mo	Striated muscle	AIDS	35	*B. vesicularum*	PYM, SDZ, ABZ, ITZ	Complete recovery, died of viral pneumonitis
9	67 y/F	United States	2004	Fever, weakness, co-infected with *Pneumocystis* sp.)	6 wk	Striated muscle	–	NA	*B. algerae*	ABZ, CLI, ATQ, ITZ	Died
10	47 y/M	Australia	2005	Weakness, co-infected with *Burkholderia pseudomallei*, MAC infections	NA	Striated muscle	AIDS	30	*T. hominis*	None	Suicide
11	67 y/F	United States	2011	Fever, tongue lesions	5 mo	Striated muscle	–	NA	*Tubulinosema acridophagus*	ABZ	Died of CLL
12	43 y/M	Thailand	2011	Fever, weakness, pulmonary hypertension	15 mo	Striated muscle, bone marrow, kidney, bladder	–	368	*Microsporidium* sp.	ABZ	Died of aspiration pneumonia

*NA, not available, –, negative; CTX, cotrimoxazole; PYM, pyrimethamine; CLI, clindamycin; ABZ, albendazole; SDZ, sulfadiazine; ITZ, itraconazole; ATQ, atovaquone; MAC*, Mycobacterium avium* complex; CLL, chronic lyphocytic leukemia.
